# Screening and Isolation of Potential Anti-Inflammatory Compounds from *Saxifraga atrata* via Affinity Ultrafiltration-HPLC and Multi-Target Molecular Docking Analyses

**DOI:** 10.3390/nu14122405

**Published:** 2022-06-09

**Authors:** Gang Li, Yan Fang, Yonggui Ma, Yangzom Dawa, Qilan Wang, Jing Gan, Jun Dang

**Affiliations:** 1 Center for Mitochondria and Healthy Aging, College of Life Sciences, Yantai University, Yantai 264005, China; ligang_ytu@126.com (G.L.); fyy2008fx@163.com (Y.F.); ganjing@ytu.edu.cn (J.G.); 2 Key Laboratory of Medicinal Animal and Plant Resources of Qinghai-Tibetan Plateau in Qinghai Province, College of Life Science, Qinghai Normal University, Xining 810001, China; 2025041@qhnu.edu.cn (Y.M.); dawayangzom@126.com (Y.D.); 3 Qinghai Provincial Key Laboratory of Tibetan Medicine Research, Key Laboratory of Tibetan Medicine Research, Chinese Academy of Sciences, Northwest Institute of Plateau Biology, Xining 810001, China; wql@nwipb.cas.cn

**Keywords:** affinity ultrafiltration, molecular docking, preparative isolation, *Saxifraga atrata*, 11-O-(4′-O-methylgalloyl)-bergenin

## Abstract

In this study, a 100 g sample of *Saxifraga atrata* was processed to separate 1.3 g of 11-O-(4′-O-methylgalloyl)-bergenin (Fr1) after 1 cycle of MCI GEL^®^ CHP20P medium pressure liquid chromatography using methanol/water. Subsequently, COX-2 affinity ultrafiltration coupled with reversed-phase liquid chromatography was successfully used to screen for potential COX-2 ligands in this target fraction (Fr1). After 20 reversed-phase liquid chromatography runs, 74.1 mg of >99% pure 11-O-(4′-O-methylgalloyl)-bergenin (Fr11) was obtained. In addition, the anti-inflammatory activity of 11-O-(4′-O-methylgalloyl)-bergenin was further validated through molecular docking analyses which suggested it was capable of binding strongly to ALOX15, iNOS, ERBB2, SELE, and NF-κB. As such, the AA metabolism, MAPK, and NF-κB signaling pathways were hypothesized to be the main pathways through which 11-O-(4′-O-methylgalloyl)-bergenin regulates inflammatory responses, potentially functioning by reducing pro-inflammatory cytokine production, blocking pro-inflammatory factor binding to cognate receptors and inhibiting the expression of key proteins. In summary, affinity ultrafiltration-HPLC coupling technology can rapidly screen for multi-target bioactive components and when combined with molecular docking analyses, this approach can further elucidate the pharmacological mechanisms of action for these compounds, providing valuable information to guide the further development of new multi-target drugs derived from natural products.

## 1. Introduction

Inflammatory responses are generally induced by inflammatory cytokines and associated inflammatory mediators [1,2]. Important inflammatory factors associated with pyrexia and related symptoms include interleukin-1 (IL-1), IL-6, and tumor necrosis factor (TNF), while pain-related inflammatory factors including prostaglandin E2 (PGE2) and bradykinin (BK), PGE2, and nitric oxide (NO) are all related to inflammation-associated vasodilation. Increases in vascular permeability in inflammatory settings are tied to the activity of histamine (HA), BK, and reactive oxygen species metabolites. Inflammatory factors associated with increased vascular permeability include histamine (HA), BK, and reactive oxygen species metabolites (ROMs), whereas oxygen free radicals, lysosomal enzymes, and NO can induce tissue damage. Notably, several signal transduction pathways coordinate the onset and termination of inflammatory responses [3,4]. As such, inhibiting the release of these inflammatory cytokines and mediators has the potential to effectively mitigate tissue damage caused during the inflammatory process. At present, antibacterial and antiviral anti-inflammatory drugs are often used in clinical treatment, while the long-term use of these drugs is prone to drug resistance and related complications. Therefore, the development of therapeutically effective and less toxic anti-inflammatory drugs is essential as a means of reliably treating inflammatory diseases in the clinic. Natural products (NPs) often serve as critical precursors in the field of drug development. Tibetan medicine is one of the most comprehensive traditional medical systems in the world [5,6,7]. The search for potential anti-inflammatory active substances derived from traditional Tibetan medicines has thus emerged as a promising approach to the development of novel drugs to prevent or treat inflammatory diseases.

*Saxifraga* L. is the largest genus in the Saxifragaceae family, with around 500 species found primarily in the circumpolar and alpine regions of the Northern Hemisphere [8]. *Saxifraga atrata* (*S. atrata*) is a member of the *Saxifraga Sect. Micranthes* and grows at elevations of 3000 to 4200 m in alpine meadows or flowstone beaches [9]. In traditional Tibetan medicine, *S. atrata* flowers are used to reduce fevers and treat lung diseases [10]. However, the morphological characteristics of different *Saxifraga* L. species are so similar that it can be difficult to differentiate them from a macroscopic perspective. Moreover, as there are limited data available regarding the active compounds present in *S. atrata*, quality control and bioactivity determination efforts remain challenging. It is thus critical that a large-scale approach to isolating and purifying standard bioactive substances from *S. atrata* be established in order to better develop its medicinal value. The complex makeup of *S. atrata*, as well as its limited solubility, have impeded the isolation of adequately pure compounds from this species to date. Silica-gel column chromatography [11] and recrystallization [12] are traditional approaches to preparing, separating, and purifying NPs. However, they are all limited by low efficiency and low recovery rates [13]. In recent years, additional extraction techniques have emerged, including high-speed counter-current chromatography (HSCCC), which separates components based upon a liquid−liquid partitioning principle based on the differential partition coefficients of different substances in two phases [14,15]. The principle of HSCCC theory is based on a hydrodynamic equilibrium system. One criterion used to judge whether the system has reached equilibrium is the observation of whether the effluent liquid was stratified. If stratification is observed, the sample can be injected into the high-speed counter-current chromatography system with separation then being achieved using a spiral tube separation based on the differential partition coefficients of the different components in the two phases, thus forming a multi-stage extraction process, with the results of this separation being recorded using a data collection system and workstation. Controlling these separation effects during the instrument’s operation necessitates a range of approaches, leading to drawbacks including poor separation resolution, the need to determine the partition coefficient, and complex operating procedures. High resolution and repeatability are essential for the separation and preparation of high-purity compounds [16,17,18]. To overcome these issues, additional separation techniques are needed to facilitate the large-scale purification of high-purity bioactive compounds derived from *S. atrata*.

Preparative high-performance liquid chromatography (prep-HPLC) has been established as an effective approach to separating a single compound from complex systems, such as biological samples and NPs. Because of its superior column efficiency, separation repeatability, online detection, and autonomous control, this technology has been extensively employed in a variety of fields [19,20]. However, to preserve the chromatographic column and simplify the subsequent separation and purification steps, prep-HPLC cannot directly separate crude extracts. Instead, target compounds must first be enriched from the crude extracts via sample pretreatment, while non-target components should be eliminated. Based on the principle of analytical liquid chromatography, which takes advantage of the fact that different substances have different partition coefficients in a system consisting of a stationary phase and a mobile phase, medium pressure liquid chromatography (MPLC) represents an effective pretreatment approach [21,22,23]. As such, the preferred approach to the large-scale preparation of high-purity bioactive compounds is a combination of MPLC and prep-HPLC [24,25]. Affinity ultrafiltration-HPLC, a high-throughput screening technique combining affinity ultrafiltration and HPLC, has emerged as a focus of growing interest owing to its ability to rapidly, efficiently, and sensitively screen for bioactive components derived from complex NPs. Affinity ultrafiltration generally includes four steps: affinity incubation, centrifugal ultrafiltration, ligand dissociation, and analysis and detection. Purified, non-covalent complexes must be collected following affinity ultrafiltration, after which appropriate methods such as the addition of organic reagents, ultrasonic treatment, or changing the pH to denature the drug target such that the small molecule ligands of interest dissociate from non-covalent complexes to permit subsequent analysis and detection [26,27].

Since its initial development in the mid-1970s, molecular docking has proven to be an important tool to help understand how compounds interact with their molecular targets, assisting in drug design and discovery [28]. Molecular docking is one of the most commonly used methods in the field of structure-based drug design, which focuses on the study of molecular interactions, as it can predict the appropriate target binding site with considerable accuracy, calculate the affinity of the resultant complex, determine the relative positions and orientations of the ligands and receptors, and thereby study the mechanisms governing the activity of a given agonist, inhibitor, or drug, laying the foundation for new drug design [29,30,31]. Molecular docking methods are invaluable in the drug research field, providing an effective tool for the discovery and optimization of lead compounds. This approach can be used to study the interaction of small molecule probes with intracellular biomolecules to identify the targets of small molecules in an organism, and to enable breakthroughs in new drug development. Based on structural biology, the structure and function of important proteins related to normal physiological processes and diseases such as cancer and inflammation can be systematically studied and analyzed to define the three-dimensional (3D) structures of protein drug targets and conduct kinetic simulation studies of drug−target protein interactions. Moreover, existing drug molecules and the active ingredients of herbal medicines can be leveraged to design new lead compounds with enhanced activity through further modification and optimization.

To the best of our knowledge, research focused on *S. atrata* to date has primarily centered on the isolation of antioxidants [20]. There has only been one report regarding the separation and purification of standard substances from this species. Briefly, using MPLC technology combining a polyamide with MCI GEL^®^ CHP20P stationary phase, Dang et al. [32] effectively isolated and purified bergenin from *S. atrata* with a purity greater than 99%. In the present study, the MCI GEL^®^ CHP20 stationary phase was a styrene−divinylbenzene matrix with exceptional hydrophobicity that was able to efficiently separate polar compounds [33,34]. First, the target component was enriched on a medium pressure chromatographic column with MCI GEL^®^ CHP20 as the stationary phase. Subsequently, a specific anti-inflammatory ingredient was effectively screened from Fr1 by the method of affinity ultrafiltration-RPLC. After that, using only one step of preparative reversed-phase liquid chromatography (prep-RPLC), a high purity sample of the anti-inflammatory active compound (11-O-(4′-O-methylgalloyl)-bergenin, Fr11) was obtained from the target fraction. Ultimately, molecular docking analyses were then used to explore interactions between this bioactive compound and the inflammatory-related targets of cyclooxygenase-2 (COX-2), 5-lipoxygenase (5-LOX), arachidonic acid 15-lipoxygenase (ALOX15), p38 mitogen-activated protein kinase (p38MAPK), c-Jun NH_2_-terminal kinase (JNK), extracellular signal-regulated kinase 5 (ERK5), epidermal growth factor receptor (EGFR), epidermal growth factor receptor 2 (ERBB2), toll-like receptors (TLR), nuclear factor kappa-B (NF-κB), TNF, tumor necrosis factor receptor 1A (TNFR1A), nitric oxide synthase (iNOS), IL-6, IL-23 receptor (IL-23R), IL-1β, IL-10, and e-selectin (SELE) in order to evaluate the anti-inflammatory activity of this sample. The predicted results suggest that the isolated potential ligand (11-O-(4′-O-methylgalloyl)-bergenin, Fr11) exhibits significant anti-inflammatory activity through multi-pathway and multi-target synergy, providing novel basis for the treatment of inflammatory diseases. Therefore, an integrated combination of affinity ultrafiltration-HPLC and multi-target molecular docking provides a powerful approach to the multi-dimensional mining of anti-inflammatory active components in *S. atrata*. This approach can be leveraged for the large-scale purification of anti-inflammatory isocoumarin standards from other NP extracts, thereby supporting the development of the NP-based pharmaceutical industry.

## 2. Materials and Methods

### 2.1. Apparatus and Chemicals 

An MPLC workstation consisting of two NP7000 prep-HPLC pumps, an NU3000 UV–Vis detector, a 5 mL manual injector, and an LC workstation was used for this study (Hanbon Science & Technology Co., Ltd., Huaian, China). HPLC analyses were conducted using an LC-16A instrument equipped with a column thermostat and autosampler following sample degassing performed using a DGU-20A3R instrument (Shimadzu Instruments Co., Shanghai, China). A Waters QDa ESI mass spectrometer (Waters Instruments Co., Milford, MA, USA) or a Q Exactive Orbitrap instrument (Thermo Fisher Scientific Inc., Waltham, MA, USA) mass spectrometer was utilized when collecting ESI-MS spectra, while ^1^H and ^13^C NMR spectra were obtained with a Bruker Avance 600 MHz (Bruker, Karlsruhe, Germany) with MeOH-*d_4_* as the solvent.

MCI GEL^®^ CHP20P (120 μm) separation materials were obtained from Mitsubishi Chemical Corporation (Tokyo, Japan). Two ReproSil-Pur C18 AQ columns (4.6 × 250 mm, 5 μm and 20 × 250 mm, 5 μm) were from Maisch Corporation (Munich, Germany). The Click XIon column (4.6 × 250 mm, 5 μm) was from ACCHROM Corporation (Beijing, China). Acetonitrile (ACN), preparative methanol (CH_3_OH), and HPLC-grade ACN were from Kelon Chemical Reagent Factory (Chengdu, China). A Moore water purification station was used to obtain ultrapure water for HPLC from deionized water (Chongqing, China). Human cyclooxygenase 2 was from Sigma-Aldrich (St. Louis, MO, USA). PBS was purchased from Solarbio Science & Technology Co. Ltd. (Beijing, China). Ultrafiltration membranes (YM-30, 30 kDa) were from Millipore Co. Ltd (Burlington, MA, USA).

### 2.2. Preparation of the Target Fraction

The *S. atrata* materials used in this study were harvested from Menyuan County, Haibei Tibetan Autonomous Prefecture, Qinghai Province (3922 m, 37°38′, 09.81″ N, 101°31′, 51.77″ E) in July 2017. The plant was identified as *S. atrata* by Qingbo Gao of the Northwest Institute of Plateau Biology of the Chinese Academy of Sciences. Medicinal material specimens are kept in the Key Laboratory of Adaptation and Evolution of Plateau Biota, Chinese Academy of Sciences (No. Gao2017080).

The harvested fresh sample was allowed to air dry under cool conditions and ground to produce a powder, after which 100 g of the resultant powder were extracted twice using methanol (4.0 L of methanol per extraction for 24 h). The obtained extract (8 L) was filtered and condensed by evaporation under decreased pressure to a volume of about 500 mL, mixed with 10 g silica gel and dried at 40 °C. Then the sample was crushed and passed through a sieve to yield a 21.8 g sample. The dried silica gel mixture was loaded into a medium pressure chromatography tower (49 × 100 mm), which was linked to a medium pressure chromatography column (49 × 460 mm) containing 1.2 L of MCI GEL CHP20P stationary phase. Elution was conducted using a methanol/water system for 0–150 min (0–100% methanol) at a 57.0 mL/min flow rate, with a 254 nm detection wavelength. This same method was repeated once, yielding an 840.0 mg sample after concentrating the target fraction with a recovery of 7.1%. 

### 2.3. Screening and Isolation of Potential Anti-Inflammatory Active Ingredients Based on Affinity Ultrafiltration-HPLC

#### 2.3.1. Screening of the Potential Anti-Inflammatory Active Ingredient in the Target Fraction with Affinity Ultrafiltration

Initially, 1 mg of the *S. atrata* fraction Fr1 was dissolved in 500 μL of PBS (pH 7.2) to prepare a 2 mg/mL sample solution for affinity ultrafiltration screening. The test sample solution (Fr1, 100 µL) was incubated with 10 µL of COX-2 (4 U) for 45 min at 37 °C in an incubator shaker. This mixture was then transferred to a 30 kDa ultrafiltration tube, centrifuged at 10,000 rpm for 10 min and washed 3 times with 200 µL of PBS to remove unbound ligands. Next, COX-2-bound ligands were released from this complex by incubation with 200 µL of methanol (90%, *v*/*v*) for 10 min at room temperature and centrifuged at 10,000 rpm for 10 min. This process was repeated in triplicate, and the filtrate was then collected, freeze-dried, and re-dissolved with 50 μL of methanol for HPLC analysis. To prepare a negative control sample, the enzyme was denatured via incubation for 10 min in boiling water, after which the operating procedures were identical to those above.

#### 2.3.2. Purification of Potential COX-2 Ligand via Reversed-Phase Liquid Chromatograph

To further purify the target sample, this 840.0 mg sample was dissolved in a 10.0 mL methanol/water (7:3, *v*/*v*) solution. It was then filtered through a 0.45 μm organic filter membrane to obtain an 84.0 mg/mL sample solution. The purification step was completed on a ReproSil-pur C18 AQ preparative column (20 × 250 mm, 5 μm), with 0.2% *v*/*v* formic acid in water as mobile phase A and chromatographic acetonitrile as mobile phase B. The injection volume was 0.5 mL and the sample was eluted with 10% acetonitrile isocratic elution at a 19 mL/min flow rate for 20 min, with 254 nm as the detection wavelength.

### 2.4. Analysis of the Purity of the Isolated Candidate COX-2 Ligand

Click XIon and ReproSil-Pur C18 AQ analytical columns were used to assess the purity of the isolated standard substance. The mobile phase A was composed of 0.2% (*v*/*v*) formic acid in water, while mobile phase B consisted of ACN. Both analytical columns were used to perform gradient elution for 30 min in 95–55% ACN and 5–50% ACN, with a flow rate of 1.0 mL/min and a 5 μL injection volume. The detection wavelength was 254 nm.

### 2.5. Molecular Docking Analyses

Molecular docking is a technique that simulates the interactions between small molecule ligands and receptor biomolecules based on the “Induced Fit Theory” of ligand−receptor interaction. By continuously optimizing the position of the small molecule compound and the dihedral angle of the flexible bond inside the molecule, the optimal conformation of the small molecule for interaction with the target macromolecule can be identified and its binding mode and affinity can be assessed, with the ligand exhibiting the highest affinity for a given receptor that is closest to its natural conformation; this is identified via a predictive scoring function. The 2D structure of the active compound was converted into a 3D structure using Chem3D 16.0. Then, the crystal structures of COX-2 (PDB ID: 5IKQ), 5-LOX (PDB ID: 3V99), ALOX15 (PDB ID: 2P0M), p38MAPK (PDB ID: 1A9U), JNK (PDB ID: 3DA6), ERK5 (PDB ID: 4IC7), EGFR (PDB ID: 2ITX), ERBB2 (PDB ID: 3PP0), TLR (PDB ID: 2Z7X), NF-κB (PDB ID: 3RZF), TNF (PDB ID: 2E7A), TNFR1A (PDB ID: 1ICH), iNOS (PDB ID: 2ORO), IL-6 (PDB ID: 1P9M), IL-23R (PDB ID: 3DUH), IL-1β (PDB ID: 5I1B), IL-10 (PDB ID: 2ILK), and SELE (PDB ID: 1GIT), were downloaded from the PDB (https://www.rcsb.org/ (accessed on 6 April 2022) database, and water molecules and free radicals were removed using the PyMOL software, followed by the addition of polar hydrogen atoms and Kollman charges. Finally, the format of the compounds and protein molecules were converted to the pdbqt format using AutoDock for molecular docking and the calculation of molecular binding energies. Minimization was performed using the Lamarckian genetic algorithm and the pseudo-Solis and Wets methods with default parameters. A total of 100 peptide conformations were defined based on docking score values, where conformations exhibiting the lowest binding energy were selected for model development. After the docking was completed, the interaction of the ligand with key amino acid residues in the active site of the protein was assessed based on the scoring results. It is generally believed that the lower the binding energy, the stronger the affinity and the better the ligand and receptor binding.

## 3. Results

### 3.1. Target Fraction MCI GEL^®^ CHP20P Medium Pressure Liquid Chromatography Sample Pretreatment

**Figure 1:**
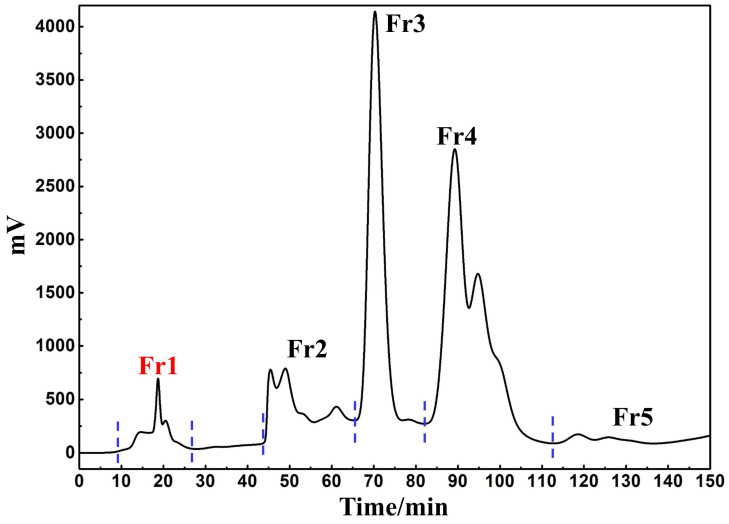
Separation chromatogram of *S. atrata* amorphous silica gel mixture. Conditions: mobile phase A: water and B: methanol; gradient: 0–150 min, 0–100% B; monitoring wavelength: 254 nm; flow rate: 57.0 mL/min; sample loading: 21.8 g of *S. atrata* amorphous silica gel combination; column temperature: room temperature.

The methanol extract of *S. atrata* contained large amounts of chlorophyll, which had the potential to interfere with the subsequent separation of the target compound and cause column contamination. It was thus necessary to eliminate it prior to any further chromatographic experiments. The MCI GEL^®^ CHP20P filler used medium pressure chromatographic separation consists of a matrix of the styrene−divinylbenzene copolymer, and polymer-based filler can maintain the stability of the spherical structures and associated properties following exposure to extreme acid−base solutions and organic solvents, thus ensuring the repeatability of target compound isolation during purification [33]. This enabled appropriate process development and optimization of separation conditions to achieve high resolution and product recovery. Based on these adsorption properties, we initially loaded 21.8 g of the mixed sample to a small medium pressure column (49 × 100 mm) and connected it to a medium pressure column (49 × 460 mm) equipped with 1.2 L of MCI GEL^®^ CHP20P for dry sample loading preparation. The separation chromatogram for this extract when using MCI GEL^®^ CHP20P and methanol−water as an eluent is shown in Figure 1. After 1 cycle, the target component was collected, concentrated, and weighed to yield 840.0 mg of sample (Fr1, recovery 7.1%). This compound was then dissolved in a methanol/water solution (7:3 *v*/*v*, 10.0 mL, 84.0 mg/mL) and filtered using a 0.45 μM organic filter membrane to facilitate further purification.

### 3.2. Screening and Isolation of Potential Anti-Inflammatory Active Ingredient Based on Affinity Ultrafiltration-HPLC

#### 3.2.1. Screening of the Potential Anti-Inflammatory Active Ingredient in the Target Fraction

**Figure 2:**
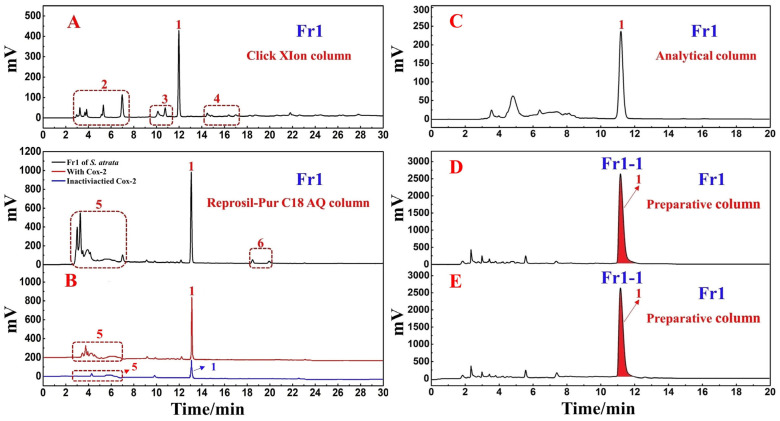
The chromatogram derived from the Click XIon analytical column (**A**) An affinity ultrafiltration-HPLC chromatogram for potential COX-2 ligands present in *S. atrata* target fraction Fr1 (**B**). The black, red, and blue lines, respectively, correspond to the HPLC chromatograms of *S. atrata* target fraction Fr1 without COX-2, with activated COX-2, and with inactivated COX-2. The analytical (**C**) and preparative (**D**,**E**) chromatograms of Fr1 on the ReproSil-Pur C18 AQ column. Number 1 represents the target main component in Fr1, while red dashed boxes 2, 3, 4, 5, and 6 highlight the impurities present in Fr1. Conditions: mobile phase A: 0.2% *v*/*v* formic acid in water, B: ACN; gradient: 0–30 min, 95–55% B for Click XIon analytical column (**A**), 0–30 min, 5–50% B for Reprosil-Pur C18 AQ analytical column (**B**) and 0–20 min, 10% B for Reprosil-Pur C18 AQ column (**C**–**E**); detection wavelength: 254 nm; flow rate: 1.0 mL/min (**A**–**C**) and 19.0 mL/min (**D,E**); injection volume: 5 μL (**A**–**C**) and 0.5 mL (**D**,**E**); column temperature: 30 °C for analysis and room temperature for preparation.

The target fraction (Fr1) was first analyzed using a Click XIon column (4.6 × 250 mm, 5 μm). The presence of a major component (peak 1) in this fraction was evident (Figure 2A), but many impurities (red dashed lines 2, 3, and 4) were also evident when using this column, and the response value was relatively low. Hydrophilic interaction chromatography (HILIC) and RPLC are known to offer effective complementary selectivity. To improve the purity of the main component (peak 1), Fr1 was re-analyzed using a ReproSil-Pur C18 AQ column (4.6 × 250 mm, 5 μm). The analytical chromatogram, shown as the black line in Figure 2B, also revealed a main component (peak 1). Observation of the black line in Figure 2B revealed that a portion of the peaks (red dotted line 5) came out in the first 5 min, possibly due to the presence of large polar substances in this fraction. Strongly polar compounds were poorly retained during reversed-phase liquid chromatography. Some impurities (red dotted line 6) were also observed behind the main peak. A comparison of the chromatographs derived from these two columns (Figure 2A,B) revealed that on the ReproSil-Pur C18 AQ analytical column, the main peak and impurities exhibited a somewhat longer peak time interval. This was more conducive to the optimization of elution conditions, allowing for the more efficient preparation of the principal component of this fraction (peak 1 in Figure 2B). When comparing the experimental conditions used for these two analytical columns, the consumption of organic reagents was reduced when using the ReproSil-Pur C18 AQ analytical column 5–50% ACN gradient elution relative to the Click XIon analytical column 95–55% ACN gradient elution during the same 30 min elution period, making the former approach more environmentally friendly. This provided further support for the different selectivity of these two analytical columns. While polar compounds were weakly retained on the RPLC analytical column such that they were not effectively separated for analysis, the HILIC approach is often used in the separation of highly polar molecules, such that these two approaches can complement one another.

To determine whether the main component in Fr1 was the potential anti-inflammatory active component of interest, we next performed a one-step affinity ultrafiltration screening step. COX-2 is an inducible isoenzyme, while COX-2 activity under basal conditions is very limited, it can be readily upregulated under inflammatory conditions, leading to an increase in PEG2, PGE1, and PGI2 production in inflammatory tissues, thus perpetuating inflammatory responses and tissue damage [35]. Affinity ultrafiltration has unique applications in the discovery of small molecule drugs due to its high sensitivity and selectivity. The magnitude of the ability of a small molecule ligand to bind to an enzyme can be expressed by the relative binding affinity (RBA). This RBA value is obtained by calculating the peak area ratio of the compound after incubation with the active and inactivated enzymes. The peak areas of the peaks were obtained from the integration of the high-performance liquid chromatograph. Compounds with an RBA greater than 1.5 are considered to be potential ligands. The RBA value for a given compound is calculated as follows:

RBA = A_S_/A_0_

where A_S_ represents the peak area of the sample incubated with the activated enzyme and A_0_ represents the peak area of the sample incubated with the inactivated enzyme. As shown in the red and blue lines in Figure 2B, the *S. atrata* Fr1 main component exhibited a good RBA value of 1.63.

#### 3.2.2. Purification of the Potential COX-2 Ligand with Reversed-Phase Liquid Chromatography

Based on the above analytical findings, a ReproSil-Pur C18 AQ preparative column (20 × 250 mm, 5 μm) was chosen for further preparation in order to increase the purity of the primary component (peak 1 in Figure 2B) while mitigating environmental harm to the greatest extent possible. The elution parameters were optimized before employing the ReproSil-Pur C18 AQ column for separation and preparation. Under isocratic elution conditions using ACN-0.2% *v*/*v* formic acid in water (0–20 min, 10% ACN), the main peak (peak 1 in Figure 2C) exhibited sufficient resolution. As a result, we conducted the following studies using these conditions. After linear amplification, the main compound (peak 1 in Figure 2D,E) was separated and purified on the ReproSil-Pur C18 AQ preparative column using the established conditions. The result of this purification step is shown in Figure 2D,E. Reproducible chromatographic separation is critically important in order to prepare a compound with the highest possible purity, minimizing the mixing of components during repeated injections. In this study, we recorded the retention time values of the target components after 10 repeated injections to assess the reproducibility of the process. As shown in Figure 2D,E, the system exhibited good reproducibility, and therefore could be used to enrich the target component via repeated collection. The sample was effectively dissolved in a 70% methanol/water solvent mixture (86.7 mg/mL), prepared with 10% ACN as the eluent at a flow rate of 19.0 mL/min and an injection volume of 0.5 mL. Fr11 was collected and concentrated after 20 replicate purification steps, yielding 74.1 mg of the sample with a recovery of 5.7%. 

### 3.3. Analyses of the Purity and Structural Characterization of Potential COX-2 Ligands

**Figure 3:**
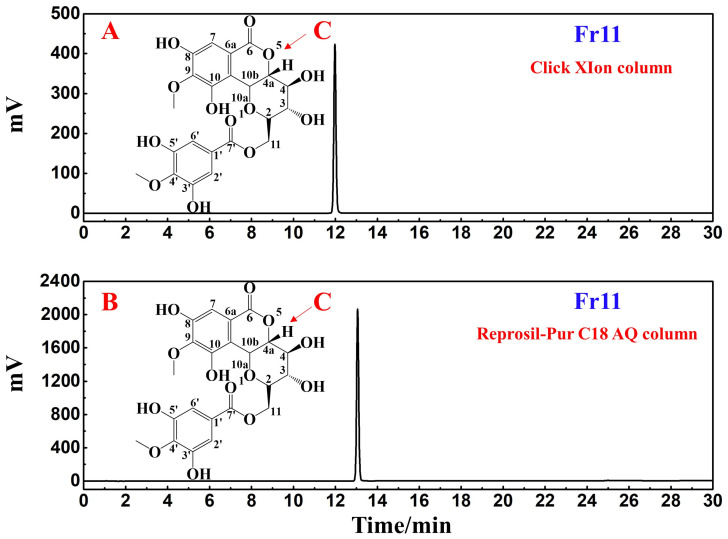
Analyses figures of the purity of the target compound in Fr11 were performed using Click XIon (**A**) and ReproSil-Pur C18 AQ (**B**) analytical columns. (**C**) shows the chemical structure of the isolated compound. Conditions: mobile phase A: 0.2% *v*/*v* formic acid in water and B: ACN; gradient: 0–30 min, 95–55% B for (**A**), 0–30 min, 5–50% B for (**B**); detection wavelength: 254 nm; flow rate: 1.0 mL/min; injection volume: 5 μL; column temperature: 30 °C.

To clarify the purity of the target compound following the above isolation procedures, different stationary phases with distinct polarities and separation mechanisms were used: one was a HILIC column (Click XIon column) and the other was a pure water-resistant RP-C18 column (ReproSil-Pur C18 AQ column). As shown in Figure 3A,B, the target compound was >99% purity, obtained for each column. RPLC relies on hydrophobic interactions between the hydrophobic stationary phase and the solute to achieve the efficient separation of weakly and moderately polar compounds. HILIC consists of a polar stationary phase and a polar mobile phase. The mobile phase used is similar to that employed for RPLC, with a weak eluent as the organic phase and a strong eluent as the aqueous phase, enabling this approach to achieve column efficiency and symmetrical peak shapes equivalent to those produced via RPLC. Thus, confirmation of the purity of the target compound Fr11 was achieved through both the Click XIon and ReproSil-Pur C18 AQ columns, with both strategies supporting the high purity of this target compound Fr11.

The obtained ESI-MS, ^1^H NMR, and ^13^C NMR spectra for the target compound in Fr11 were compared with published literature to clarify the structural characteristics of this compound (Figure 3C). The structural identification of Fr11 based on these data is shown in Supplementary Figures S1–S3, with all of these data (summarized below) supporting the identity of this target compound as 11-O-(4′-O-methylgalloyl)-bergenin.

Compound Fr11 (11-O-(4′-O-methylgalloyl)-bergenin, 74.1 mg, white powder, ESI-MS *m*/*z*: 493.32 [M-H]^-^, calc. for C_22_H_22_O_13_ *m*/*z* 494.1060): ^1^H NMR (600 MHz, MeOH-d_4_) 7.09 (2H, s, H-2′, 6′), 7.08 (1H, s, H-7), 5.03 (1H, d, J = 10.5 Hz, H-10b), 4.87 (H, dd, J = 5.2, 4.2 Hz, H-11a), 4.41 (1H, dd, J = 11.2, 6.7 Hz, H-11b), 4.10 (1H, dd, J = 10.5, 9.5 Hz, H-4a), 3.97 (1H, m, H-2), 3.90 (3H, s, C-9-OCH_3_), 3.86 (3H, s, C-4′-OCH_3_), 3.85 (1H, m, H-4), 3.35 (1H, s, H-3); ^13^C NMR (151 MHz, MeOH-d_4_) 167.8 (C-4′), 165.7 (C-6), 152.4 (C-8), 151.9 (C-3′, 5′), 149.4 (C-10), 142.4, (C-9) 141.5 (C-7′), 126.2 (C-1′), 119.5 (C-6a), 117.1 (C-10a), 111.2 (C-7), 110.4 (C-2′, 6′), 81.4 (C-4a), 80.6 (C-2), 75.5 (C-4), 74.5 (C-10b), 71.9 (C-3), 62.0 (C-11), 61.0 (C-9-OCH_3_), 60.8 (C-4′-OCH_3_). These ESI-MS, ^1^H NMR, and ^13^C NMR spectra thus revealed this compound to be 11-O-(4′-O-methylgalloyl)-bergenin based on a comparison with published literature sources [36].

### 3.4. Molecular Docking Analyses of the Interactions between 11-O-(4′-O-methylgalloyl)-bergenin and Inflammation-Related Targets 

**Figure 4:**
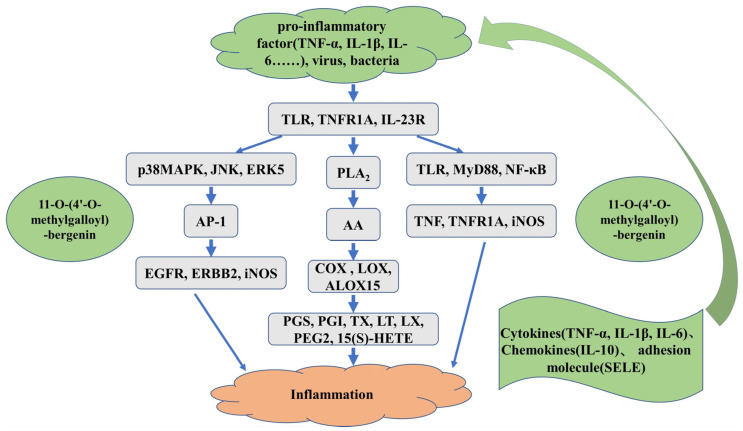
The schematic diagram of the involved inflammatory-related mechanisms.

Although we successfully screened and isolated candidate COX-2 ligand from *S. atrata* via affinity ultrafiltration-HPLC, the mechanistic basis for the predicted ligand−enzyme interactions remained unclear. To further explore the role of this potential anti-inflammatory active derivative of *S. atrata* (11-O-(4′-O-methylgalloyl)-bergenin, Fr11), a molecular docking method was used to study the binding mode and binding energy of 11-O-(4′-O-methylgalloyl)-bergenin when interacting with inflammation-related targets. Inflammatory responses are shaped through interactions between various inflammatory and anti-inflammatory factors that regulate the biological effects of inflammatory cells through different signal transduction pathways, with different inflammatory cell types, cytokines, adhesion molecules, and inflammatory mediators, all cooperating to determine the characteristics of a given inflammatory response. After reviewing the relevant literature [37,38], a molecular docking analysis was carried out for common inflammatory signaling pathways including arachidonic acid (AA) metabolism, MAPK, and TLR/myeloid differentiation factor 88 (MyD88)/NF-κB pathways. Figure 4 illustrated a summary of the involved inflammatory-related mechanisms in the molecular docking analysis.

#### 3.4.1. AA Metabolic Pathway

Phospholipase A_2_ (PLA_2_) leases AA from the membrane phospholipid, whereupon it can be used to produce prostaglandins, prostacyclins, thromboxanes, leukotrienes, lipoxins, arachidonic acid ethanolamine, and epoxyeicosatrienoic acids via the COX and LOX pathways [35]. These metabolites affect neutrophil recruitment and infiltration, platelet aggregation, epithelial barrier function, vascular permeability and bronchoconstriction, in addition to inducing inflammatory responses. The ALOX15 protease is a heme iron-free dioxygenase that catalyzes and esterifies the peroxidation of polyunsaturated fatty acids and produces a series of bioactive lipid intermediates. Notably, ALOX15 is involved in early inflammatory responses and its metabolite 15-hydroxyeicosatetraenoic acid (15(S)-HETE) is a potent pro-inflammatory chemoattractant for neutrophils and leukocytes. 

**Figure 5:**
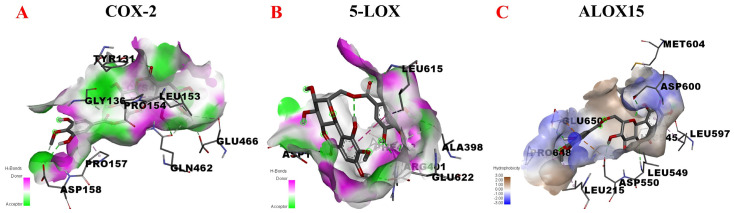
Molecular docking analysis of the putative binding between 11-O-(4′-O-methylgalloyl)-bergenin and COX-2, 5-LOX, and ALOX15. (**A**–**C**) correspond to the binding models for interactions between 11-O-(4′-O-methylgalloyl)-bergenin and COX-2, 5-LOX, and ALOX15, respectively. Hydrophobic or H-bond interactions are displayed as a colored surface.

Given the above considerations, COX-2, 5-LOX, and ALOX15 were selected for molecular docking with 11-O-(4′-O-methylgalloyl)-bergenin (Fr11) to assess its potential anti-inflammatory activity. These docking results are shown in Figure 5. When docking with COX-2, 11-O-(4′-O-methylgalloyl)-bergenin (Fr11) was located in the pocket surrounded by multiple amino acids (LEU-153, TYR-131, PRO-154, GLU-466, GLN-462, HIS-39, GLY-136, CYS-47, CYS-36, PRO-157, and ASP-158), interacting through van der waals, conventional hydrogen bond, carbon hydrogen bond, and pi-alkyl acting force interactions (Figure 5A). When docking with 5-LOX, 11-O-(4′-O-methylgalloyl)-bergenin (Fr11) was located in a pocket surrounded by ASP-170, GLU-622, ARG-401, PHE-402, LEU-615, and ALA-398, interacting through conventional hydrogen bond and pi-sigma and pi-alkyl acting force interactions (Figure 5B). When docking with ALOX15, 11-O-(4′-O-methylgalloyl)-bergenin (Fr11) was located in a pocket surrounded by LEU-597, HIS-545, LEU-549, ASP-600, MET-640, ASP-550, LEU-215, and GLU-650, interacting through conventional hydrogen bond, carbon hydrogen bond, and pi-anion and pi-alkyl acting force interactions (Figure 5C). The binding of COX-2 and 5-LOX to 11-O-(4′-O-methylgalloyl)-bergenin (Fr11) was dominated by hydrogen bonding (Figure 5A,B), while for ALOX15, this binding was dominated by hydrophobic interactions (Figure 5C). As shown in Table 1, the binding energy values for interactions between 11-O-(4′-O-methylgalloyl)-bergenin (Fr11) and 5-LOX, COX-2, and ALOX15 in this study were −3.66 kcal/mol, −6.35 kcal/mol, and −9.36 kcal/mol, respectively. Overall, these results suggested that the docking of 11-O-(4′-O-methylgalloyl)-bergenin (Fr11) with 5-LOX was not stable, such that 5-LOX is unlikely to represent a target of the anti-inflammatory activity of this compound. In contrast, the docking binding energy for the interaction between COX-2 and 11-O-(4′-O-methylgalloyl)-bergenin (Fr11) of −6.35 kcal/mol largely supported the high RBA value (1.63) detected via affinity ultrafiltration-HPLC. In addition, 11-O-(4′-O-methylgalloyl)-bergenin (Fr11) exhibited a binding energy of −9.36 kcal/mol when interacting with ALOX15, binding to amino acids in 2P0M primarily through hydrophobic interactions and thus reducing the energy of the system, forming a stable structure. Thus, we can predict that 11-O-(4′-O-methylgalloyl)-bergenin (Fr11) is likely to exert its anti-inflammatory effects mainly through the COX-2 pathway by modulating AA metabolism, with such activity also being closely related to ALOX15. 

#### 3.4.2. MAPK Signaling Pathway

The MAPK is an important signaling pathway that plays a critical role in mediating diverse cellular responses, leveraging a highly conserved three-level kinase cascade to transmit signals. Extracellular stimuli activate MAPK kinase kinase (MKKK) proteins, which in turn activate MAPK kinase (MKK), leading to the activation of MAPK proteins via the dual phosphorylation of tyrosine and threonine [38]. JNK, p38 MAPK, and ERK5 are the major subfamilies of MAPK. JNK, also known as stress-activated protein kinase (SAPK), is functionally similar to p38 MAPK, as both can be activated by various inflammatory cytokines and play important roles in stress responses related to conditions such as inflammation and apoptosis [39]. ERK5 mainly regulates cell growth and differentiation and is mediated by the upstream Ras/Raf signal protein, transmitting stimulatory signals to the nucleus and thereby regulating the proliferation, differentiation, and apoptosis of macrophages [40]. EGFR can bind to receptor tyrosine kinase (RTK) and when EGFR expression is enhanced, it can induce epithelial growth factor to form a complex with RTK through the Ras/Raf/MAPK signaling pathway, influencing epithelial cell proliferation and differentiation in a manner that can drive inflammatory activity. ERBB2 is a 185 kDa cell membrane receptor encoded by the proto-oncogene erbB-2. ERBB2 phosphorylation also causes ERBB2 to activate the Ras/Raf/MAPK signaling pathway [41]. 

**Figure 6:**
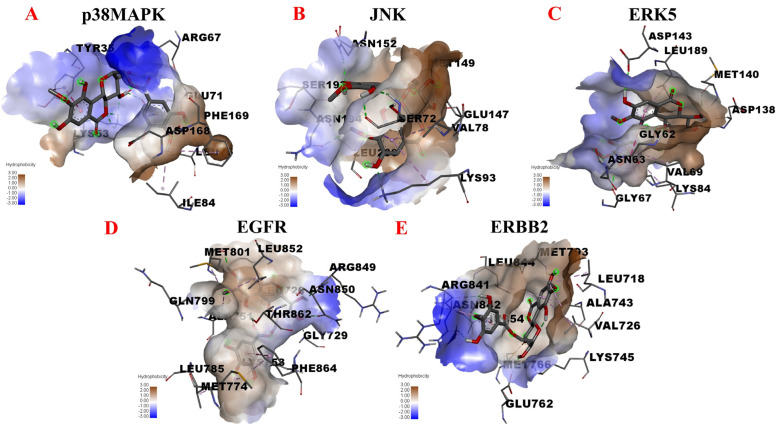
Molecular docking analyses of the putative binding between 11-O-(4′-O-methylgalloyl)-bergenin and p38 MAPK, JNK, ERK5, EGFR, and ERBB2. (**A**–**E**) correspond to the binding models for interactions between 11-O-(4′-O-methylgalloyl)-bergenin and p38 MAPK, JNK, ERK5, EGFR, and ERBB2, respectively. Hydrophobic interactions are displayed as a colored surface.

In light of the above information, we next assessed potential interactions between 11-O-(4′-O-methylgalloyl)-bergenin (Fr11) and p38MAPK, JNK, ERK5, EGFR, and ERBB2 through molecular docking analyses. The results are shown in Figure 6. When docking with p38 MAPK, 11-O-(4′-O-methylgalloyl)-bergenin (Fr11) was predicted to bind to ASP-168, GLU-71, LYS-53, ILE-84, PHE-169, LEU-75, and TYR-35 in 1A9U, interacting through conventional hydrogen bond, pi-alkyl, pi-anion and pi-pi stacked acting force (Figure 6A). When docking with JNK, 11-O-(4′-O-methylgalloyl)-bergenin (Fr11) was predicted to bind to VAL-78, LYS-93, LEU-206, GLU-147, ASN-194, SER-72, SER-193, and ASN-152 in 3DA6, interacting through conventional hydrogen bond, carbon hydrogen bond, pi-sigma and pi-alkyl acting force (Figure 6B). When docking with ERK5, 11-O-(4′-O-methylgalloyl)-bergenin (Fr11) was predicted to bind to LEU-189, ASP-143, MET-140, ASN-63, GLY-62, ASP-138, GLY-67, VAL-69, and LYS-84 through van der waals, conventional hydrogen bond, pi-cation, amide-pi stacked and pi-alkyl acting force (Figure 6C). When docking with EGFR, 11-O-(4′-O-methylgalloyl)-bergenin (Fr11) was predicted to bind to ARG-841, ASN-842, THR-854, GLU-762, LEU-718, LYS-745, VAL-726, MET-766, MET-793, LEU-844, and ALA-743, interacting through conventional hydrogen bond, sulfur-x, and pi-alkyl acting force interactions (Figure 6D). When docking with ERBB2, 11-O-(4′-O-methylgalloyl)-bergenin (Fr11) was predicted to bind to ALA-751, LEU-852, LEU-726, MET-801, ARG-849, GLN-799, ASN-850, THR-862, GLY-729, LYS-753, MET-774, PHE-864, and LEU-785, interacting through conventional hydrogen bond, carbon hydrogen bond, pi-sigma, pi-pi t-shaped, and pi-alkyl acting force interactions (Figure 6E). As such, 11-O-(4′-O-methylgalloyl)-bergenin (Fr11) was able to access the binding sites of p38MAPK, JNK, ERK5, EGFR, and ERBB2, binding to these sites primarily through hydrophobic interactions (Figure 6A–E). The binding energy values calculated for the interaction between this compound and p38MAPK, JNK, ERK5, EGFR, and ERBB2 were −6.21 kcal/mol, −6.87 kcal/mol, −6.31 kcal/mol, −6.51 kcal/mol, and −8.35 kcal/mol, respectively (Table 2). As all of these values were below −5.0 kcal/mol, this was considered indicative of good binding activity. It was thus hypothesized that 11-O-(4′-O-methylgalloyl)-bergenin (Fr11) exerts its anti-inflammatory activity by reducing the release of pro-inflammatory mediators including leukotriene (LTs) and TNF-α, primarily via interactions with the p38MAPK, JNK, ERK5, EGFR and ERBB2 components of the MAPK signaling pathway, with the inhibition of ERBB2 being the most robust, suggesting that this may be an important mechanism whereby it can regulate inflammatory responses.

#### 3.4.3. TLR/MyD88/NF-κB Pathway

The TLR/MyD88/NF-κB pathway is an important signaling pathway that regulates inflammatory responses. TLR proteins are innate immune receptors, with TLR2 and TLR4 playing particularly central roles in inflammatory responses. MyD88 is a key molecule in the TLR signaling pathway and is involved in the initiation of inflammation through its role in the transduction of upstream signal information. TLR2 and TLR4 can bind to MyD88 after engaging their cognate ligands, thereby activating the downstream transcription factor NF-κB and driving the upregulation of inflammatory mediators, including IL-1β, IL-6, TNF-α, and other inflammatory cytokines, resulting in an inflammatory cascade response [42]. As a core transcriptional regulator of inflammation, NF-κB is rapidly activated in response to pathogens and in the context of immune system activation, while the inhibition of the NF-κB pathway can effectively treat many inflammatory diseases [43]. Blocking the NF-κB signaling pathway can inhibit the high expression of iNOS and reduce the levels of it and inflammatory mediators including NO, IL-6, and TNF-α, thereby exerting anti-inflammatory effects [44]. The TNF signaling pathway also plays a role in the induction of systemic inflammatory responses and the acute phase response. TNF can trigger the activation of many pathways, including the NF-κB and MAPK pathways, and can be separated into two structurally distinct isoforms (TNF-α and TNF-β). Macrophages secrete large quantities of TNF-α after foaming when activated, in turn promoting NF-κB pathway activation, downregulating the expression of iNOS downstream of NF-κB and thereby attenuating the local inflammatory response [45]. TNFR1A is a type I TNFR that mediates inflammatory responses mainly through NF-κB signaling, the induction of apoptosis, and the promotion of IL-6 secretion [46]. There are three different isoforms of iNOS, including endothelial nitric oxide synthase (eNOS), which is expressed under normal physiological conditions, neuronal nitric oxide synthase (nNOS) and iNOS, which is induced following organismal injury where upon it can promote the production of large amounts of NO. Excessive NO production can result in acute or chronic inflammation and given its role as the most important mediator of NO production, iNOS is an important regulator of the overall inflammatory response [47]. 

**Figure 7:**
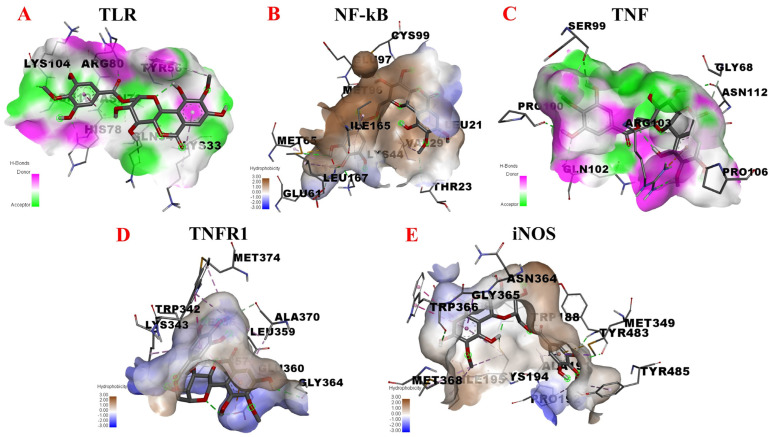
Molecular docking analyses of the putative binding between 11-O-(4′-O-methylgalloyl)-bergenin and TLR, NF-κB, TNF, TNFR1A, and iNOS. (**A**–**E**) correspond to the binding models for interactions between 11-O-(4′-O-methylgalloyl)-bergenin and TLR, NF-κB, TNF, TNFR1A, and iNOS, respectively. Hydrophobic or H-bond interactions are displayed as a colored surface.

Given the above, we assessed potential interactions between 11-O-(4′-O-methylgalloyl)-bergenin (Fr11) and TLR, NF-κB, TNF, TNFR1A, and iNOS via a molecular docking approach. When docking with TLR, 11-O-(4′-O-methylgalloyl)-bergenin (Fr11) was predicted to bind to a multiple amino acid pocket surrounded (ASN-103, LYS-104, HIS-78, ASN-79, ARG-80, GLN-54, TYR-56, and LYS-33) by conventional hydrogen bond, carbon hydrogen bond, amide-pi stacked, and pi-alkyl acting force interactions (Figure 7A). When docking with NF-κB, 11-O-(4′-O-methylgalloyl)-bergenin (Fr11) was predicted to bind to a pocket surrounded by LEU-21, CYS-99, VAL-29, THR-23, MET-65, GLU-97, ILE-165, LYS-44, MET-96, and GLU-61 via conventional hydrogen bond, carbon hydrogen bond, pi-sigma, pi-sulfur, and pi-alkyl acting force interactions (Figure 7B). When docking with TNF, 11-O-(4′-O-methylgalloyl)-bergenin (Fr11) was predicted to bind to a pocket surrounded by PRO-100, GLN-102, SER-99, ARG-103, PRO-106, ASN-112, and GLY-68 through conventional hydrogen bond, carbon hydrogen bond, and pi-cation acting force interactions (Figure 7C). When docking with TNFR1A, 11-O-(4′-O-methylgalloyl)-bergenin (Fr11) was predicted to bind to a pocket surrounded by GLY-364, ASP-357, GLU-360, LYS-343, LEU-359, MET-374, TRP-342, and ALA-370 through conventional hydrogen bond, carbon hydrogen bond, pi-anion, pi-pi stacked and pi-alkyl acting force interactions (Figure 7D). When docking with iNOS, 11-O-(4′-O-methylgalloyl)-bergenin (Fr11) was predicted to bind to a pocket surrounded by MET-368, ILE-195, GLY-365, GLN- 199, ASN-364, TRP-366, CYS-194, TYR-483, TRP-188, ALA-191, MET-349, PRO-192, and TYR-485 through van der waals, conventional hydrogen bond, pi-cation, amide-pi stacked and pi-alkyl acting force interactions (Figure 7E). As shown in Table 3, the respective binding energy values for interactions between 11-O-(4′-O-methylgalloyl)-bergenin (Fr11) and iNOS and NF-κB were −9.30 kcal/mol, −7.33 kcal/mol, respectively, with both being less than −7.0 kcal/mol, consistent with a strong binding interaction. Moreover, the binding energy values for interactions between 11-O-(4′-O-methylgalloyl)-bergenin and TNF, TNFR1A, and TLR were −5.62 kcal/mol, −5.87 kcal/mol, and −5.42 kcal/mol, respectively, with these values being below −5.0 kcal/mol, consistent with good bonding stability. Given these results and the associated signaling pathways, these data suggest that 11-O-(4′-O-methylgalloyl)-bergenin (Fr11) may regulate inflammatory responses primarily by reducing the production of pro-inflammatory cytokines and inhibiting the expression of key proteins that initiate inflammatory responses.

#### 3.4.4. Inflammation-Associated Factors and Adhesion Molecule

Interleukins (ILs) are an important class of inflammatory factors, with at least 38 ILs having been identified that play important roles in the regulation of the maturation, activation, and proliferation of immune cells. The regulation of the levels of specific IL family proteins is a key indicator used to evaluate the anti-inflammatory efficacy of many drugs. Here, we selected four representative inflammatory factors for molecular docking analyses exploring the mechanistic basis for potential anti-inflammatory activity [48]. IL-6 belongs to a family of glycoproteins that vary in molecular weight size from 26-30 kDa due to cell-specific post-translational modifications. IL-6 participates in immune regulation, hematopoietic, and inflammatory processes. Macrophages can secrete IL-6 in response to pathogen-associated molecular patterns (PAMPs), which induce an intracellular signaling cascade response, which leads to the production of inflammatory cytokines. IL-23R is a receptor subunit specific for IL-23, which acts as a pro-inflammatory factor that induces macrophages to produce LTs and TNF-α influencing tissue-specific autoimmune inflammation. IL-1β is a cytokine that plays an important role in regulating, inflammatory responses, and mediating the activation, proliferation, and differentiation of T cells and B cells. IL-1β is thought to be associated with macrophage foam cell formation. IL-10 can be produced and secreted by a variety of immune cells such as macrophages and B cells whereupon it functions as an important anti-inflammatory factor that can negatively regulate immune negative responses and maintain inflammatory homeostasis. SELE is a cell surface glycoprotein that mediates cell−cell and cell−extracellular matrix adhesion. SELE upregulation is a key component of the initiation of the inflammatory response. During inflammatory responses, leukocytes can extravasate from the plasma through adhesion to vascular endothelial cells, in turn contributing to tissue edema and further inflammation [49]. 

**Figure 8:**
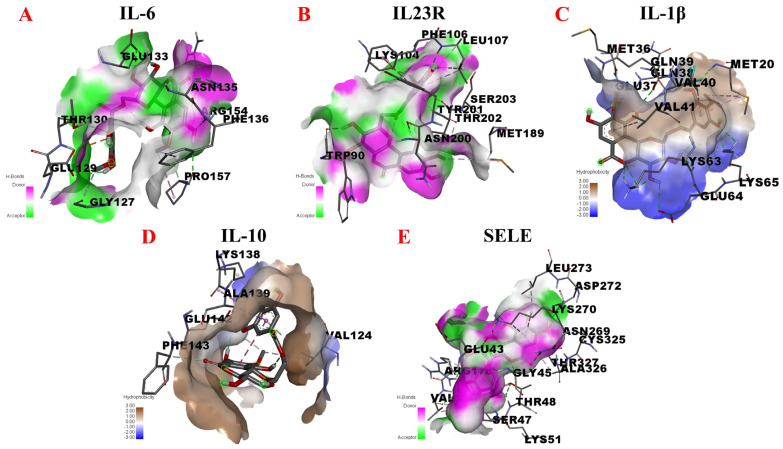
Molecular docking analysis of the putative binding between 11-O-(4′-O-methylgalloyl)-bergenin and IL-6, IL-23R, IL-1β, IL-10, and SELE. (**A**–**E**) correspond to the binding models for interactions between 11-O-(4′-O-methylgalloyl)-bergenin and IL-6, IL-23R, IL-1β, IL-10, and SELE, respectively. Hydrophobic or H-bond interactions are displayed as a colored surface.

Given the above, inflammatory factors (IL-6, IL-23R, IL-1β and IL-10) and an adhesion molecule (SELE) were selected in this study to further explore the potential anti-inflammatory targets of 11-O-(4′-O-methylgalloyl)-bergenin (Fr11), as shown in Figure 8. When docking with IL-6, 11-O-(4′-O-methylgalloyl)-bergenin (Fr11) was predicted to bind to PHE-136, ARG-154, ASN-135, PRO-157, GLU-133, THR-130, GLY-127, and GLU-129 in 1P9M via conventional hydrogen bond, carbon hydrogen bond, pi-anion, pi-alkyl and pi-sigma acting force (Figure 8A). When docking with IL-23R, 11-O-(4′-O-methylgalloyl)-bergenin (Fr11) was predicted to bind to LYS-104, SER-203, PHE-106, LEU-107, TYR-201, ASN-200, THR-202, TRP-90, and MET-189 in 3DUH via conventional hydrogen bond, carbon hydrogen bond, pi-donor hydrogen bond, pi-pi t-shaped and pi-alkyl acting force (Figure 8B). When docking with IL-1β, 11-O-(4′-O-methylgalloyl)-bergenin (Fr11) was predicted to bind to GLU-64, LYS-63, VAL-40, MET-20, GLN-38, VAL-41, LYS-65, GLU-37, GLN-39, and MET-36 in 5I1B via conventional hydrogen bond, carbon hydrogen bond and pi-alkyl acting force (Figure 8C). When interacting with IL-10, 11-O-(4′-O-methylgalloyl)-bergenin (Fr11) was predicted to bind to PHE-143, VAL-124, GLU-142, ALA-139, and LYS-138 sites via conventional hydrogen bond, carbon hydrogen bond, pi-alkyl, pi-sigma and amide-pi stacked acting force (Figure 8D). When docking with SELE, 11-O-(4′-O-methylgalloyl)-bergenin (Fr11) was predicted to bind to CYS-325, THR-327, ASN-269, ALA-326, ASP-272, LEU-273, LYS-270, GLY-45, ARG-178, GLU-43, THR-48, VAL-179, SER-47, THR-177, and LYS-51, interacting through conventional hydrogen bond, pi-sigma, pi-lone pair, sulfur-x and pi-alkyl acting force interactions (Figure 8E). Binding between this compound and IL-6, IL-23R, and SELE was dominated by hydrogen bonding (Figure 8A,B,E), whereas its interactions with IL-1β and were dominated by hydrophobic interactions (Figure 8C,D). As shown in Table 4, the binding energy values for interactions between 11-O-(4′-O-methylgalloyl)-bergenin (Fr11) and IL-6, IL-23R, IL-1β, and IL-10 were −5.62 kcal/mol, −6.20 kcal/mol, −5.52 kcal/mol and −4.02 kcal/mol, respectively, whereas the binding energy for SELE was −7.93 kcal/mol. Lower binding energy values generally correspond to more stable interactions, with values below −5.0 kcal/mol and −7.0 kcal/mol, corresponding to good and strong binding activity, respectively. As such, we speculate that 11-O-(4′-O-methylgalloyl)-bergenin (Fr11) may exert its anti-inflammatory activity primarily via inhibiting the production of pro-inflammatory cytokines and the expression of the adhesion molecule, rather than by promoting the release of anti-inflammatory cytokines.

The inflammatory response is a complex process that involves multiple genes and signaling pathways. By combining the above analysis results with target and pathway analyses, we can predict that 11-O-(4′-O-methylgalloyl)-bergenin (Fr11), a bioactive component of *S. atrata* isolated via affinity ultrafiltration-HPLC, may exert anti-inflammatory effects through four mechanisms: blocking the binding of pro-inflammatory factors to their cognate receptors, inhibiting the expression of key proteins that initiate the inflammatory response, reducing the production of pro-inflammatory cytokines, and regulating cell proliferation so as to indirectly regulate the inflammatory response. As such, this study provides a convenient means of exploring the mechanisms of interaction between inflammation-related targets and *S. atrata*-derived ligands, guiding the future development of anti-inflammatory active components from *S. atrata*.

## 4. Discussion

Uncertainty regarding the main components of NPs is a problem that is commonly encountered when attempting to establish quality control standards for these products. As such, the ability to isolate high-purity standard compounds from complex NPs is critical to the development of the NPs industry as a whole. NPs are also important precursors for the design of novel anti-inflammatory drugs. Inflammation is a physiological response to adverse stimuli and associated damage, and can cause cellular degeneration, necrosis, and abnormal metabolic activity [50]. 

Nonsteroidal anti-inflammatory drugs (NSAIDs) are a broad class of anti-inflammatory agents. Since aspirin was first synthesized in 1898, more than 100 types of NSAIDs have been marketed under thousands of brand names, including aspirin, acetaminophen, indomethacin, ibuprofen, and rofecoxib [51]. They are widely used in clinical practice for the treatment of rheumatoid arthritis, fevers, and pain. However, prolonged NSAID use can result in the emergence of drug resistance and associated complications [52]. There is thus an urgent need to screen for novel anti-inflammatory drugs. Molecular docking approaches offer significant technical advantages as a means of evaluating medicinal compounds and their putative pharmacological targets, offering a means of more accurately screening and clarifying potential pharmacodynamic and pharmacological mechanisms of action for drugs of interest.

Accordingly, in this study, a one-step MCI GEL^®^ CHP20P MPLC approach was successfully performed to pretreat *S. atrata* samples, after which a one-step affinity ultrafiltration-RPLC strategy was used to screen for potential COX-2 ligand in the target fraction Fr1. Subsequent preparation RPLC was then used to isolate 11-O-(4′-O-methylgalloyl)-bergenin (Fr11), a potent anti-inflammatory active ingredient that was >99% pure. These results thus demonstrate the feasibility of specifically isolating COX-2 ligands with potential anti-inflammatory activity from *S. atrata*. To explore the binding ability of 11-O-(4′-O-methylgalloyl)-bergenin (Fr11) when interacting with inflammation-related targets, molecular docking analyses were conducted that revealed it to bind to the following compounds, which are ranked in descending order based on predicted binding energy values: ALOX15, iNOS, ERBB2, SELE, NF-κB, JNK, EGFR, COX-2, ERK5, p38MAPK, IL-23R, TNFR1A, TNF, IL-6, IL-1β, TLR, IL-10, and 5-LOX. 

This study is the first to our knowledge to use a molecular docking approach to report on the potential anti-inflammatory mechanism of action of the *S. atrata*-derived active ingredient 11-O-(4′-O-methylgalloyl)-bergenin (Fr11), revealing this compound exhibits a high degree of binding activity towards ALOX15, iNOS, ERBB2, SELE, and NF-κB. Combined target and pathway analyses thus suggested that 11-O-(4′-O-methylgalloyl)-bergenin (Fr11) may primarily regulate inflammatory responses through the targeting of the AA metabolism, MAPK, and NF-κB signaling pathways, exerting its anti-inflammatory activity through four primary mechanisms: blocking the binding between pro-inflammatory factors and their cognate receptors, inhibiting the expression of key proteins that initiate the inflammatory response, reducing the production of pro-inflammatory cytokines, and regulating cellular proliferation so as to indirectly regulate the inflammatory response. A key advantage of this study is that it provides a sound theoretical basis for further research and development focused on the anti-inflammatory effects of isocoumarins such as 11-O-(4′-O-methylgalloyl)-bergenin (Fr11), revealing novel directions for research exploring the multi-component and multi-target modes of action of traditional Chinese medicinal compounds.

It is important to note that molecular docking techniques are constantly being updated, that some data are uncertain, and that the predicted results need to be validated by future cellular, animal-based, and clinical trials. These results suggest that the prioritization of mechanistic studies exploring pathways associated with ALOX15, iNOS, ERBB2, and SELE may of particular relevance for efforts to clarify the molecular basis for the activity of 11-O-(4′-O-methylgalloyl)-bergenin. For example, flow cytometry can be utilized as a means of assessing reactive oxygen species production and mitochondrial transmembrane potential, whereas western blotting can be used to evaluate the expression of proteins of interest. PCR can be used to assess mitochondrial copy numbers, thus offering comprehensive insight regarding the mechanism of action for this *S. atrata*-derived compound as an anti-inflammatory mediator. This approach will provide a robust experimental foundation for the design of new anti-inflammatory drugs.

## 5. Conclusions 

The medicinal efficacy of NPs is primarily attributable to the complex chemical characteristics of their secondary metabolites, which can synergistically interact with multiple drug targets or multiple biochemical pathways to exert their overall efficacy. In this study, an MCI GEL^®^ CHP20P medium pressure column with a methanol−water mobile phase was used for the effective enrichment of the methanol extract of *S. atrata* and the target fraction (Fr1) was successfully prepared. By comparing the separation effects of Click XIon and ReproSil-Pur C18 AQ analytical columns for this target fraction (Fr1), a ReproSil-Pur C18 AQ preparative column was selected for subsequent separation and purification. COX-2 affinity ultrafiltration coupled with reversed-phase liquid chromatography was successfully used to screen for candidate COX-2 ligands in this target fraction (Fr1). The preparative isolation of this potential COX-2 ligand was then completed via isocratic elution in 10% ACN, and an HPLC purity analysis conducted using HILIC and RP-18 columns confirmed that the sample (74.1 mg) collected in 20 replicate cycles was 11-O-(4′-O-methylgalloyl)-bergenin and that it was >99% pure. To further validate the anti-inflammatory effect of the thus putative COX-2 ligand, molecular docking techniques were used to predict its anti-inflammatory targets. This comprehensive analytical approach revealed that can readily bind to ALOX15, iNOS, ERBB2, and SELE, providing a theoretical justification for future justification assays. Overall, these results and future validation efforts will provide a basis for the discovery of additional drugs derived from *S. atrata* and other medicinal plants that can be used to treat various inflammatory diseases based upon correlations between bioactive compounds and their putative biological targets. 

## Figures and Tables

**Table 1:** Intermolecular interactions between Fr11 and COX-2, 5-LOX, and ALOX15.

Proteins	Protein Function	Binding Energy (kcal/mol)	Binding Residues	Type
COX-2	Inflammation, pain	−6.35	LEU-153 TYR-131 PRO-154 GLU-466 GLN-462 HIS-39 GLY-136 CYS-47 CYS-36 PRO-157 ASP-158	Pi-Alkyl Conventional Hydrogen Bond Pi-Alkyl Carbon Hydrogen Bond Conventional Hydrogen Bond van der Waals Carbon Hydrogen Bond van der Waals Carbon Hydrogen Bond Pi-Alkyl Conventional Hydrogen Bond
5-LOX	Inflammation, pain	−3.66	ASP-170 GLU-622 ARG-401 PHE-402 LEU-615 LEU-615 ALA-398	Conventional Hydrogen Bond Conventional Hydrogen Bond Conventional Hydrogen Bond Pi-Alkyl Conventional Hydrogen Bond Pi-Sigma Conventional Hydrogen Bond
ALOX15	Inflammation, immunity, neuroprotection	−9.36	LEU-597 HIS-545 HIS-545 LEU-549 ASP-600 MET-640 ASP-550 ASP-550 LEU-215 GLU-650 GLU-650	Pi-Alkyl Carbon Hydrogen Bond Conventional Hydrogen Bond Conventional Hydrogen Bond Conventional Hydrogen Bond Pi-Alkyl Conventional Hydrogen Bond Pi-Anion Pi-Alkyl Conventional Hydrogen Bond Pi-Anion

**Table 2:** Intermolecular interactions between Fr11 and p38 MAPK, JNK, ERK5, EGFR, and ERBB2.

Proteins	Protein Function	Binding Energy (kcal/mol)	Binding Residues	Type
p38 MAPK	Inflammation, apoptosis, proliferation, differentiation	−6.21	ASP-168 GLU-71 GLU-71 LYS-53 ILE-84 PHE-169 LEU-75 TYR-35 TYR-35	Conventional Hydrogen Bond Pi-Alkyl Pi-Anion Conventional Hydrogen Bond Pi-Pi Stacked Pi-Alkyl Pi-Alkyl Pi-Pi Stacked Pi-Alkyl
JNK	Inflammation, apoptosis, proliferation, differentiation	−6.87	VAL-78 LYS-93 LYS-93 LEU-206 LEU-206 GLU-147 ASN-194 SER-72 SER-72 SER-193 ASN-152	Pi-Sigma Conventional Hydrogen Bond Pi-Alkyl Pi-Sigma Pi-Alkyl Conventional Hydrogen Bond Carbon Hydrogen Bond Conventional Hydrogen Bond Carbon Hydrogen Bond Conventional Hydrogen Bond Conventional Hydrogen Bond
ERK5	proliferation, differentiation, development	−6.31	LEU-189 ASP-143 MET-140 ASN-63 GLY-62 ASP-138 GLY-67 VAL-69 LYS-84	Pi-Alkyl Conventional Hydrogen Bond Conventional Hydrogen Bond van der Waals Amide-Pi Stacked Conventional Hydrogen Bond Conventional Hydrogen Bond Pi-Alkyl Pi-Cation
EGFR	Inflammation, proliferation, differentiation	−6.51	ARG-841 ARG-841 ASN-842 THR-854 GLU-762 LEU-718 LYS-745 VAL-726 MET-766 MET-793 LEU-844 ALA-743	Conventional Hydrogen Bond Pi-Alkyl Conventional Hydrogen Bond Conventional Hydrogen Bond Conventional Hydrogen Bond Pi-Alkyl Conventional Hydrogen Bond Pi-Alkyl Sulfur-X Conventional Hydrogen Bond Pi-Alkyl Pi-Alkyl
ERBB2	Inflammation, proliferation	−8.35	ALA-751 LEU-852 LEU-726 MET-801 MET-801 ARG-849 GLN-799 ASN-850 THR-862 GLY-729 LYS-753 MET-774 PHE-864 PHE-864 LEU-785	Pi-Alkyl Pi-Sigma Pi-Sigma Conventional Hydrogen Bond Pi-Alkyl Conventional Hydrogen Bond Carbon Hydrogen Bond Conventional Hydrogen Bond Conventional Hydrogen Bond Carbon Hydrogen Bond Pi-Alkyl Pi-Alkyl Pi-Alkyl Pi-Pi T-Shaped Pi-Alkyl

**Table 3:** Intermolecular interactions between Fr11 and TLR, NF-κB, TNF, TNFR1A, and iNOS.

Proteins	Protein Function	Binding Energy (kcal/mol)	Binding Residues	Type
TLR	Inflammation, immunity, survival, proliferation	−5.42	ASN-103 LYS-104 HIS-78 ASN-79 ARG-80 GLN-54 TYR-56 LYS-33	Carbon Hydrogen Bond Carbon Hydrogen Bond Conventional Hydrogen Bond Amide-Pi Stacked Conventional Hydrogen Bond Conventional Hydrogen Bond Conventional Hydrogen Bond Pi-Alkyl
NF-κB	Inflammation, immunity	−7.33	LEU-21 LEU-21 CYS-99 VAL-29 THR-23 MET-65 MET-65 MET-65 GLU-97 ILE-165 LYS-44 LYS-44 MET-96 GLU-61	Conventional Hydrogen Bond Pi-Sigma Conventional Hydrogen Bond Pi-Alkyl Carbon Hydrogen Bond Conventional Hydrogen Bond Pi-Alkyl Pi-Sulfur Conventional Hydrogen Bond Pi-Alkyl Conventional Hydrogen Bond Pi-Alkyl Pi-Alkyl Carbon Hydrogen Bond
TNF	Fever, proliferation, differentiation, inflammation, cytotoxicity	−5.62	PRO-100 GLN-102 SER-99 SER-99 ARG-103 ARG-103 PRO-106 ASN-112 GLY-68	Conventional Hydrogen Bond Conventional Hydrogen Bond Conventional Hydrogen Bond Carbon Hydrogen Bond Conventional Hydrogen Bond Pi-Cation Carbon Hydrogen Bond Conventional Hydrogen Bond Carbon Hydrogen Bond
TNFR1A	Inflammation, apoptosis	−5.87	GLY-364 ASP-357 GLU-360 GLU-360 LYS-343 LYS-343 LEU-359 MET-374 TRP-342 TRP-342 ALA-370	Conventional Hydrogen Bond Conventional Hydrogen Bond Conventional Hydrogen Bond Pi-Anion Conventional Hydrogen Bond Pi-Alkyl Pi-Alkyl Pi-Alkyl Pi-Alkyl Pi-Pi Stacked Carbon Hydrogen Bond
iNOS	Inflammation, apoptosis	−9.30	MET-368 ILE-195 GLY-365 GLN-199 ASN-364 TRP-366 TRP-366 CYS-194 CYS-194 TYR-483 TRP-188 ALA-191 MET-349 PRO-192 TYR-485	Pi-Alkyl Pi-Alkyl Pi-Sigma Conventional Hydrogen Bond Conventional Hydrogen Bond Conventional Hydrogen Bond Pi-Sulfur Conventional Hydrogen Bond Pi-Alkyl Conventional Hydrogen Bond Carbon Hydrogen Bond Pi-Sigma Conventional Hydrogen Bond Pi-Alkyl Pi-Alkyl

**Table 4:** Intermolecular interactions between Fr11 and IL-6, IL-23R, IL-1β, and IL-10.

Proteins	Protein Function	Binding Energy (kcal/mol)	Binding Residues	Type
IL-6	Inflammation, immunity, hematopoiesis	−5.62	PHE-136 PHE-136 ARG-154 ASN-135 PRO-157 PRO-157 GLU-133 THR-130 GLY-127 GLU-129	Conventional Hydrogen Bond Pi-Alkyl Pi-Sigma Conventional Hydrogen Bond Conventional Hydrogen Bond Pi-Alkyl Conventional Hydrogen Bond Carbon Hydrogen Bond Carbon Hydrogen Bond Conventional Hydrogen Bond Pi-Anion
IL-23R	Inflammation, immunity	−6.20	LYS-104 SER-203 PHE-106 LEU-107 LEU-107 LEU-107 TYR-201 ASN-200 THR-202 TRP-90 MET-189	Conventional Hydrogen Bond Carbon Hydrogen Bond Pi-Pi T-Shaped Conventional Hydrogen Bond Pi-Alkyl Pi-Lone Pair Carbon Hydrogen Bond Conventional Hydrogen Bond Carbon Hydrogen Bond Conventional Hydrogen Bond Pi-Alkyl
IL-1β	Inflammation, immunity, proliferation, differentiation	−5.52	GLU-64 LYS-63 VAL-40 MET-20 MET-20 GLN-38 VAL-41 LYS-65 GLU-37 GLN-39 MET-36	Conventional Hydrogen Bond Conventional Hydrogen Bond Pi-Alkyl Conventional Hydrogen Bond Pi-Alkyl Conventional Hydrogen Bond Pi-Alkyl Conventional Hydrogen Bond Conventional Hydrogen Bond Conventional Hydrogen Bond Carbon Hydrogen Bond
IL-10	Inflammation, immunity, apoptosis	−4.02	PHE-143 VAL-124 GLU-142 ALA-139 LYS-138 LYS-138	Carbon Hydrogen Bond Pi-Alkyl Conventional Hydrogen Bond Pi-Sigma Amide-Pi Stacked Pi-Alkyl
SELE	Inflammation	−7.93	CYS-325 THR-327 ASN-269 ALA-326 ASP-272 ASP-272 LEU-273 LYS-270 LYS-270 GLY-45 ARG-178 ARG-178 GLU-43 THR-48 VAL-179 SER-47 THR-177 LYS-51	Sulfur-X Pi-Sigma Conventional Hydrogen Bond Pi-Alkyl Conventional Hydrogen Bond Pi-Alkyl Pi-Alkyl Conventional Hydrogen Bond Pi-Sigma Conventional Hydrogen Bond Conventional Hydrogen Bond Pi-Lone Pair Conventional Hydrogen Bond Conventional Hydrogen Bond Conventional Hydrogen Bond Conventional Hydrogen Bond Pi-Lone Pair Conventional Hydrogen Bond

## Data Availability

All data generated or analyzed during this study are included in this published article (and its Supplementary Materials file).

## Supplementary Materials

The following supporting information can be downloaded at: https://www.mdpi.com/article/10.3390/nu14122405/s1, Figure S1: Schematic of the study design; Figure S2: Actual micro gel chp20p medium pressure liquid chromatography system; Figure S3: Schematic diagram of the principle of affinity ultrafiltration-HPLC; Figure S4: ESI mass spectrum of 11-O-(4′-O-methylgalloyl)-bergenin; Figure S5: ^1^H NMR spectrum (600 MHz) of 11-O-(4′-O-methylgalloyl)-bergenin (in MeOH-*d*_4_); Figure S6: ^13^C NMR spectrum (151 MHz) of 11-O-(4′-O-methylgalloyl)-bergenin (in MeOH-*d*_4_).
