# Supplementary material for: Screening and Isolation of Potential Anti-Inflammatory Compounds from Saxifraga atrata via Affinity Ultrafiltration-HPLC and Multi-Target Molecular Docking Analyses
Source: Nutrients. 2022 Jun 9;14(12):2405. doi: 10.3390/nu14122405 (PMC9230087; doi:10.3390/nu14122405)
Supplement: Supplementary file 1 [file nutrients-14-02405-s001.zip › nutrients-1738540-supplementary.pdf]

nutrients

Supplementary Materials

Screening and Isolation of Potential Anti-Inflammatory Compounds from *Saxifraga atrata* via Affinity Ultrafiltration-HPLC and Multi-Target Molecular Docking Analyses

Gang Li [1], Yan Fang [1], Yonggui Ma [2], Yangzom Dawa [2], Qilan Wang [3], Jing Gan [1] and Jun Dang [3,*]

Table of Contents

Figure S1. Schematic of the study design 2

Figure S2. Actual micro gel chp20p medium pressure liquid chromatography system 3

Figure S3. Schematic diagram of the principle of affinity ultrafiltration-HPLC 4

Figure S4. ESI mass spectrum of 11-O-(4'-O-methylgalloyl)-bergenin 5

Figure S5. 1H NMR Spectrum (600 MHz) of 11-O-(4'-O-methylgalloyl)-bergenin (in MeOH-d4) 6

Figure S6. 13C NMR Spectrum (151 MHz) of 11-O-(4'-O-methylgalloyl)-bergenin (in MeOH-d4) 7

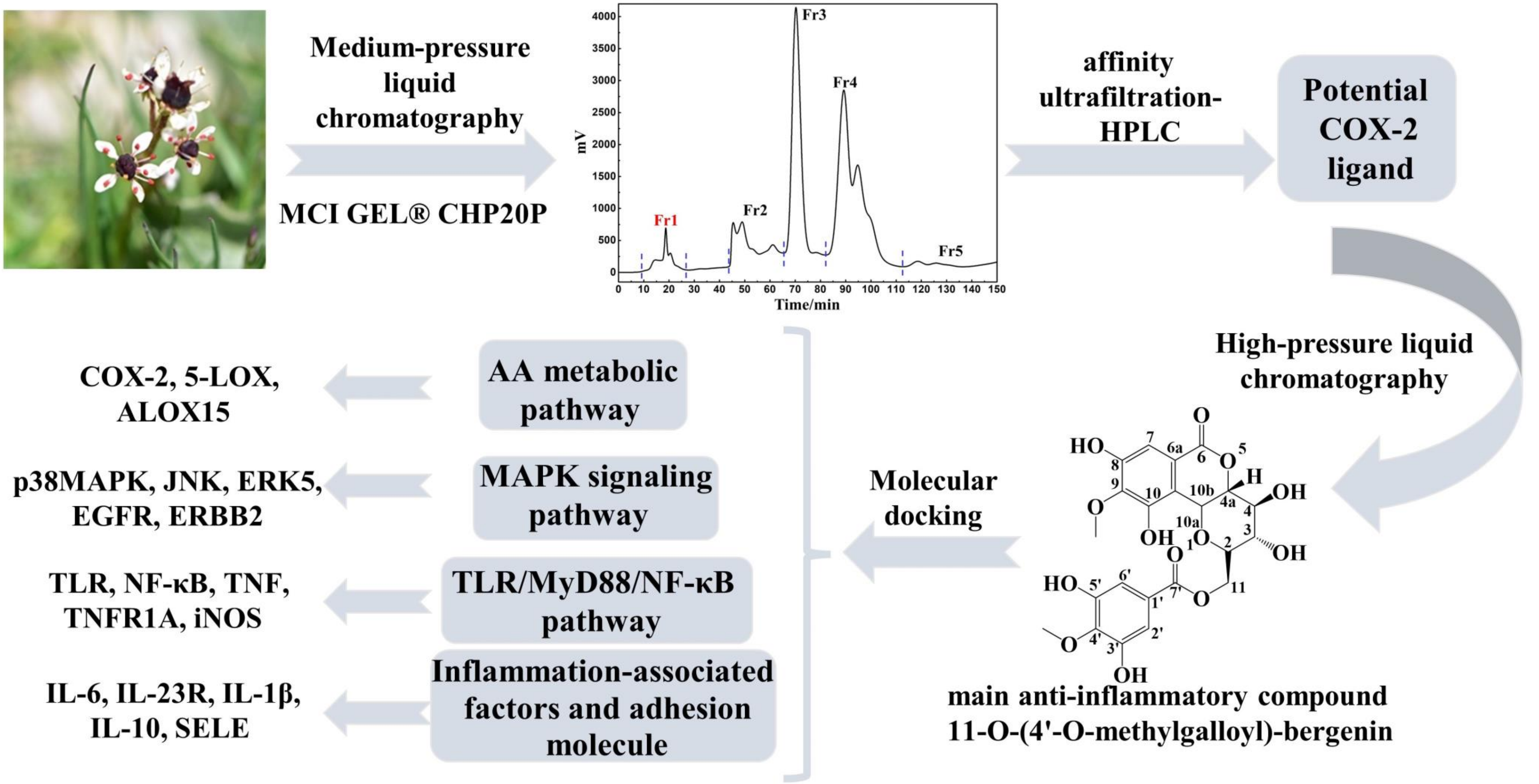

Figure S1. Schematic of the study design.

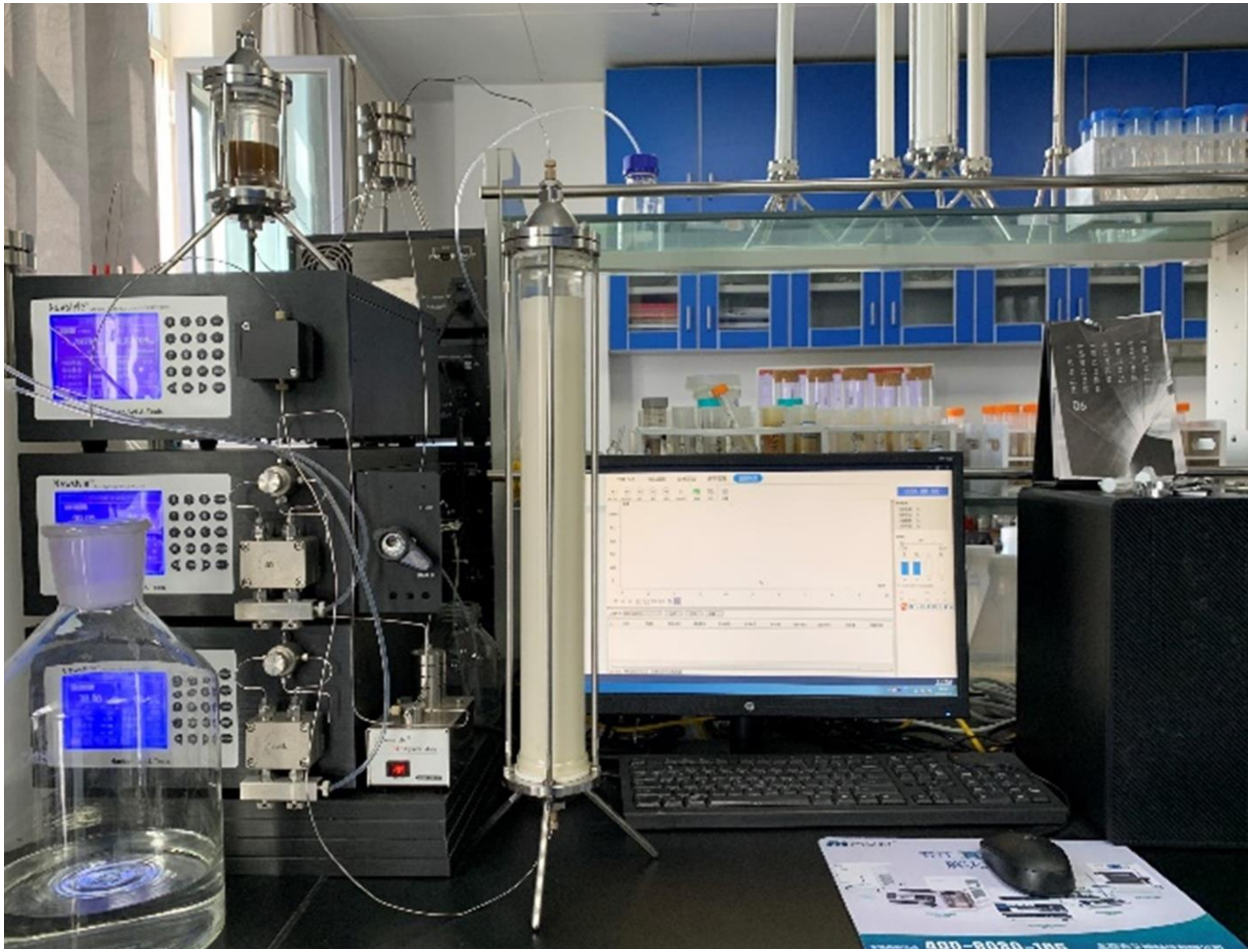

Figure S2. Actual micro gel chp20p medium pressure liquid chromatography system.

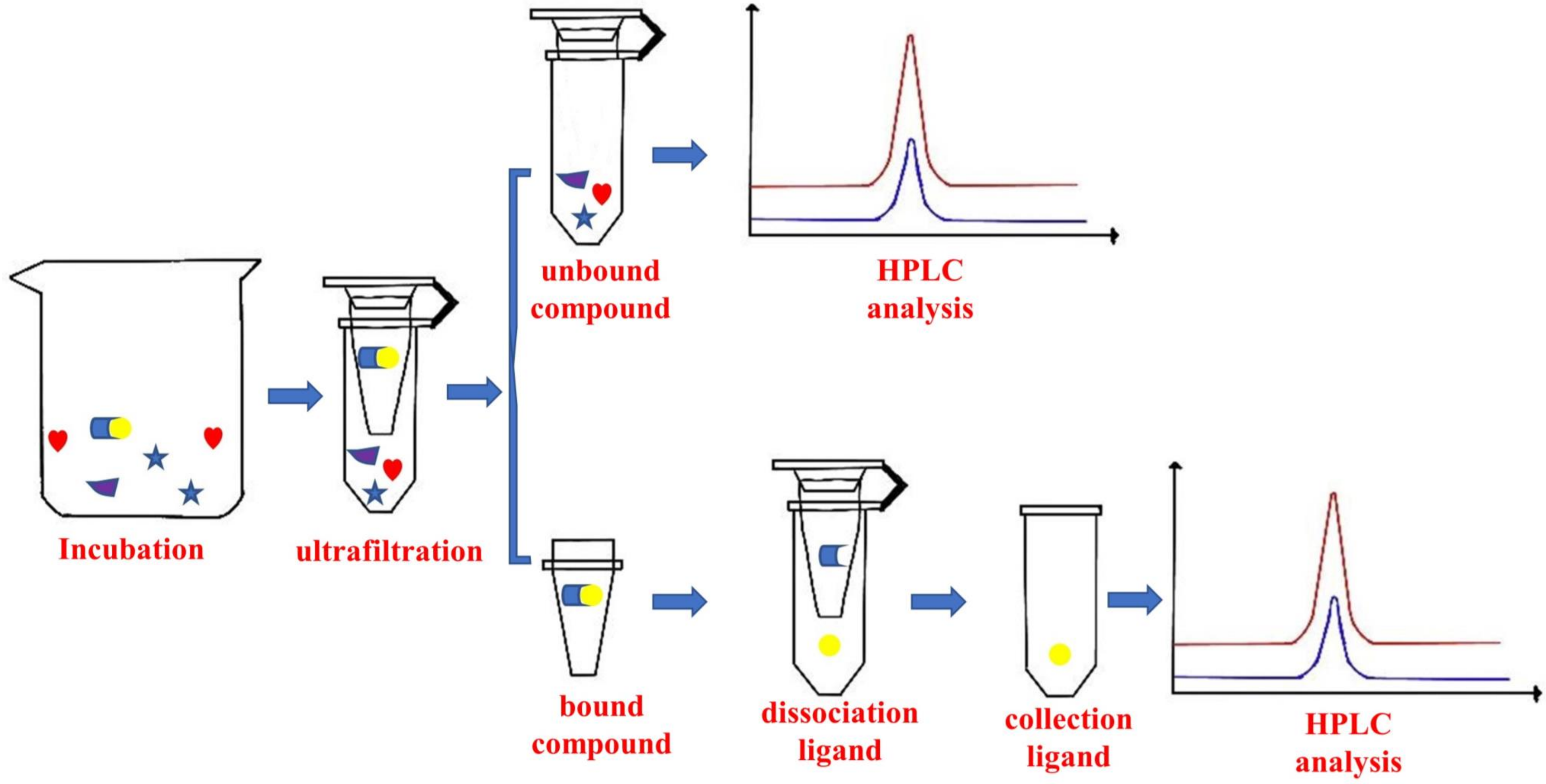

Figure S3. Schematic diagram of the principle of affinity ultrafiltration-HPLC.

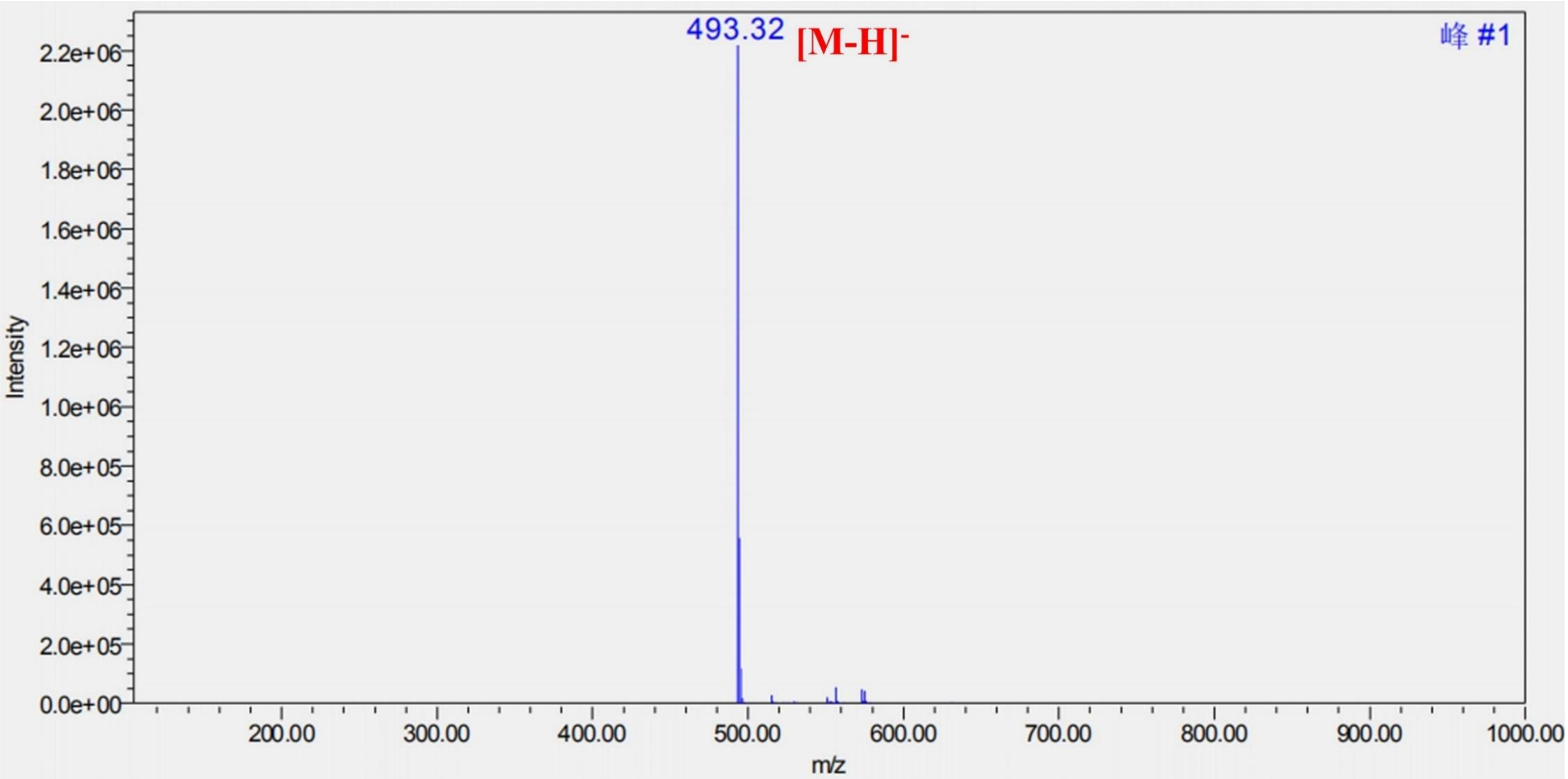

Figure S4. ESI mass spectrum of 11-O-(4'-O-methylgalloyl)-bergenin.

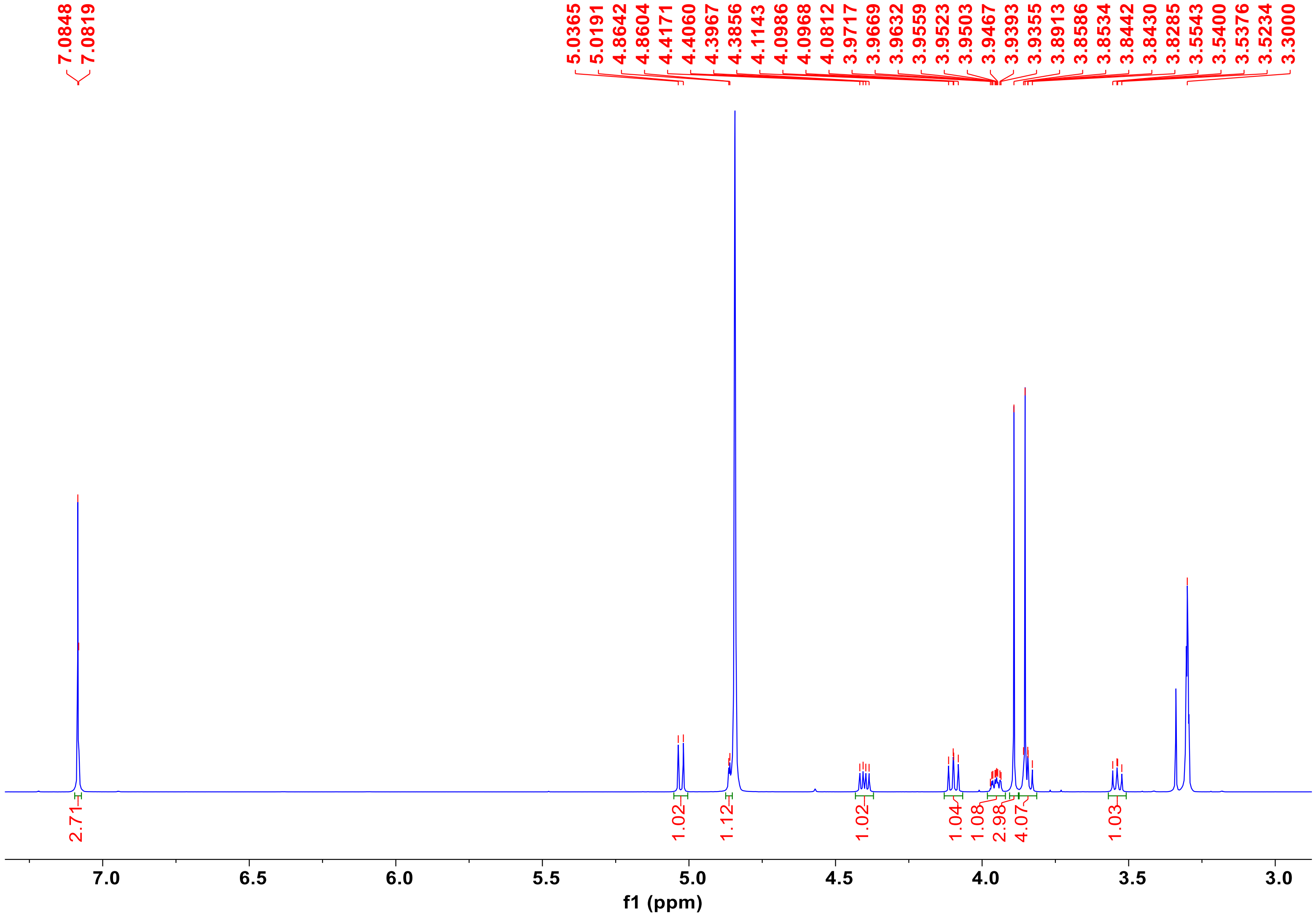

Figure S5. ^{1}H NMR Spectrum (600 MHz) of 11-O-(4'-O-methylgalloyl)-bergenin (in MeOH-d_4).

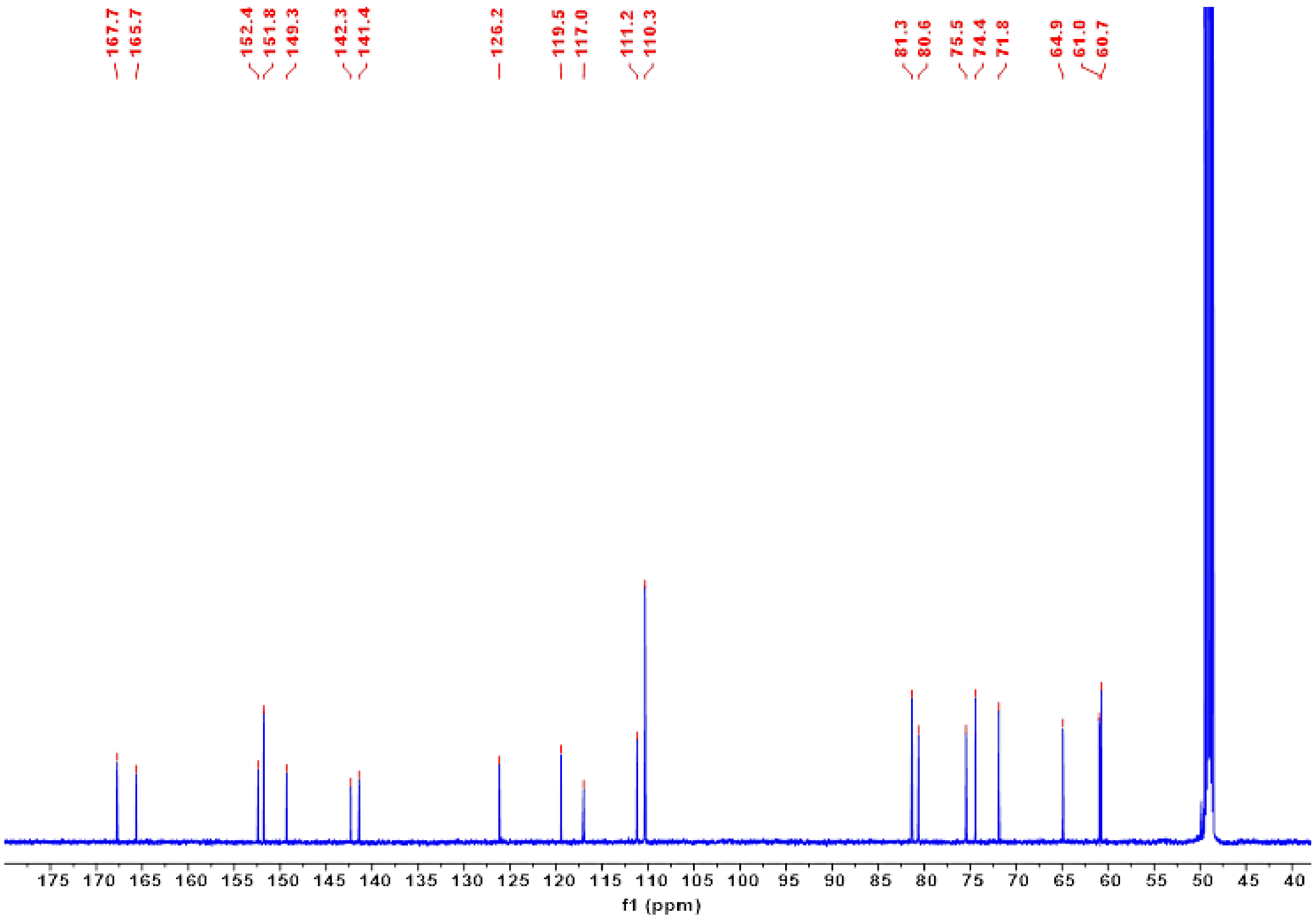

Figure S6. ^{13}C NMR Spectrum (151 MHz) of 11-O-(4'-O-methylgalloyl)-bergenin (in MeOH-d_4).
